# Exosomes Derived from Adipose Mesenchymal Stem Cells Promote Regeneration of Injured Liver in Minipigs

**DOI:** 10.3390/ijms25126604

**Published:** 2024-06-15

**Authors:** Yue Wang, Chenxi Piao, Tao Liu, Xiangyu Lu, Yajun Ma, Jiantao Zhang, Haiyang Ma, Hongbin Wang

**Affiliations:** College of Veterinary Medicine, Northeast Agricultural University, Harbin 150030, China; wangyuemooney@163.com (Y.W.); pcx7001@163.com (C.P.); liutaotiger@163.com (T.L.); lxy1997202206@163.com (X.L.); mayajun1994@163.com (Y.M.); zhangjiantao@neau.edu.cn (J.Z.); hyma5925@163.com (H.M.)

**Keywords:** mesenchymal stem cells, exosomes, hepatectomy, ischemia reperfusion, minipig, liver regeneration

## Abstract

Hepatic ischemia/reperfusion injury (IRI) is an important factor affecting liver regeneration and functional recovery postoperatively. Many studies have suggested that mesenchymal stem cells (MSCs) contribute to hepatic tissue repair and functional recovery through paracrine mechanisms mediated by exosomes. Minipigs exhibit much more similar characteristics of the liver to those of humans than rodents. This study aimed to explore whether exosomes from adipose-derived MSCs (ADSCs-exo) could actively promote liver regeneration after hepatectomy combined with HIRI in minipigs and the role they play in the cell proliferation process. This study also compared the effects and differences in the role of ADSCs and ADSCs-exo in the inflammatory response and liver regeneration. The results showed that ADSCs-exo suppressed histopathological changes and reduced inflammatory infiltration in the liver; significantly decreased levels of ALT, TBIL, HA, and the pro-inflammatory cytokines TNF-α, IL-6, and CRP; increased levels of the anti-inflammatory cytokine IL-10 and the pro-regeneration factors Ki67, PCNA, CyclinD1, HGF, STAT3, VEGF, ANG1, ANG2; and decreased levels of the anti-regeneration factors SOCS3 and TGF-β. These indicators above showed similar changes with the ADSCs intervention group. Indicating that ADSCs-exo can exert the same role as ADSCs in regulating inflammatory responses and promoting liver regeneration. Our findings provide experimental evidence for the possibility that ADSCs-exo could be considered a safe and effective cell-free therapy to promote regeneration of injured livers.

## 1. Introduction

Ischemia/reperfusion injury (IRI) refers to the cellular injury caused by the restoration of blood supply to a tissue or organ following a period of ischemia, which not only fails to reinstate the original functionality of the ischemic site but instead exacerbates the damage caused by the ischemia [1]. During liver resection, it is challenging to prevent or manage hepatic IRI, caused by interruptions in hepatic blood flow [2]. After liver resection, the remaining tissue is exposed to increased blood flow shear and metabolic stress. While proper shear force can promote the recovery of liver function and the regeneration of liver cells, insufficient or excessive shear force can damage the tissue [3]. However, IRI decreases the tolerance of residual hepatic tissue to the hemodynamic shear forces [4], which eventually impacts postoperative liver regeneration and functional recovery and may culminate in liver failure and even injury to distant organs postoperatively [5]. Furthermore, although orthotopic liver transplantation is currently the most effective therapeutic approach for end-stage liver disease [6], the graft also inevitably undergoes IRI during the transplantation procedure, significantly impacting post-transplant hepatic function and survival [7]. Therefore, comprehension of the mechanisms underlying hepatic IRI is imperative to developing effective preventive and therapeutic strategies and promoting liver regeneration.

Mesenchymal stem cells (MSCs) are known as “seed cells” in tissue engineering and regenerative medicine due to their strong self-renewal capacity and their differentiation potential into multiple lineages [8]. Additionally, numerous studies have shown that MSCs can promote tissue repair through paracrine effects [9,10,11,12,13,14], especially in the form of exosomes [15,16,17,18,19], which are important mediators of intercellular signal transmission [20]. The ability of MSC-derived exosomes (MSCs-exo) to promote liver regeneration has been demonstrated in rodents [21]. However, the results from rodent studies have certain limitations, greatly differ from clinical findings, and are primarily relevant to basic research [22,23]. In contrast, large-animal models are relatively more suitable for comparative medical research [24]. Unfortunately, there is no report on the role of MSCs-exo in liver regeneration in large animals, presumably due to the difficult management and high cost.

Adipose-derived MSCs (ADSCs) are considered promising MSCs due to the availability of source tissues and less invasive acquisition methods. We have previously demonstrated that the ADSCs-secretome has a similar effect as ADSCs in promoting liver regeneration in minipigs [25]. However, it remains unknown whether MSCs-exo, as a critical active component of the MSCs-secretome, can participate in the repair and regeneration of liver injury in large animals. Therefore, here, we used minipigs that underwent a minimally invasive laparoscopic liver partial resection combined with IRI to investigate the role of ADSCs-exo in promoting liver regeneration and, at the same time, compare the differences in effect and mechanisms with ADSCs.

## 2. Results

### 2.1. ADSCs-exo Improved the Histopathological Changes after Liver Injury

**Figure 1:**
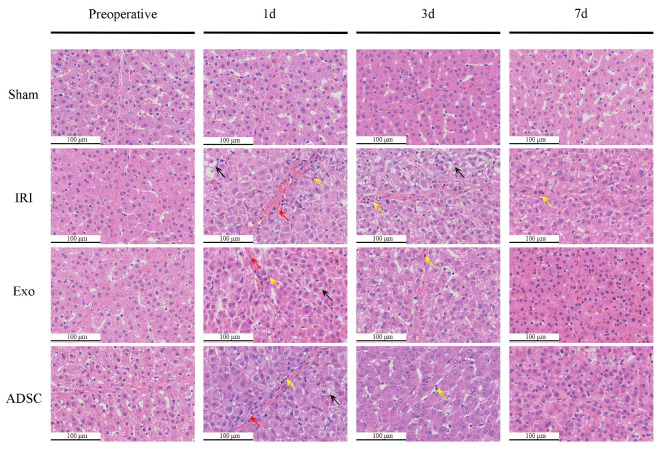
Histopathological changes in the liver. The black arrow indicates hepatocyte swelling; the red arrow indicates hemorrhage; and the yellow arrow indicates inflammatory cells.

The pathological changes in the liver tissues were examined by HE staining, as illustrated in the images in Figure 1. On the first day after liver injury, the IRI group displayed disrupted hepatic cord structures, a lack of liver sinusoids, varying degrees of hemorrhage and inflammatory cell infiltration in the interlobular spaces, and significant hepatocyte swelling and vacuolar degeneration. In contrast, the hepatic cord structures and liver sinusoids were relatively intact in the Exo and ADSC groups, and only mild hepatocyte swelling, vacuolar changes, and inflammatory infiltration were observed. On the third day after surgery, the degree of hepatocyte swelling and inflammatory infiltration was reduced in all groups. However, the IRI group still exhibited disorganized hepatic cord structures and unclear liver sinusoid architecture, as opposed to the relatively restored tissue structure in the Exo and ADSC groups. After 7 days, the hepatic cord and sinusoidal structures were almost restored in the IRI group, although mild hepatocyte swelling and inflammatory infiltration persisted. In contrast, the Exo and ADSC groups showed almost complete restoration, with a noticeable increase in hepatocyte nuclear density and rearrangement. In summary, ADSCs and ADSCs-exo interventions ameliorated liver injury induced by IRI, leading to improved hepatic tissue structure and hepatocyte recovery compared to that in the untreated IRI group.

### 2.2. ADSCs-exo Improved Liver Function after Injury

**Figure 2:**
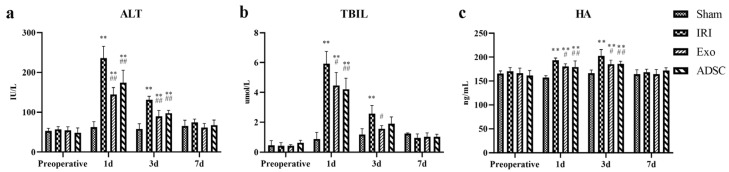
The ALT (**a**), TBIL (**b**), and HA (**c**) levels in the serum. The results are presented as mean ± SD, ** *p* < 0.01 versus the Sham group, ^#^ *p* < 0.05, ^##^ *p* < 0.01 versus the IRI group.

The serum levels of ALT, TBIL, and HA were measured to evaluate the extent of hepatocellular and sinusoidal endothelial cell damage and their functional recovery (Figure 2). ALT, TBIL, and HA levels increased significantly after surgery (1d, 3d: *p* < 0.01), and ALT and TBIL peaked on day 1 post-operation, while HA peaked on day 3 post-operation. The Exo and ADSC groups showed significantly lower levels of all three indicators compared to the IRI group on days 1 and 3 post-operation (*p* < 0.01 or *p* < 0.05). TBIL was normalized on day 3 in the Exo and ADSC groups (*p* > 0.05 vs. Sham group), which was earlier than that in the IRI group. These results indicated that ADSCs and ADSCs-exo interventions can effectively alleviate liver injury and promote the recovery of liver function in minipigs.

### 2.3. ADSCs-exo Modulated the Inflammatory Balance after Liver Injury

**Figure 3:**
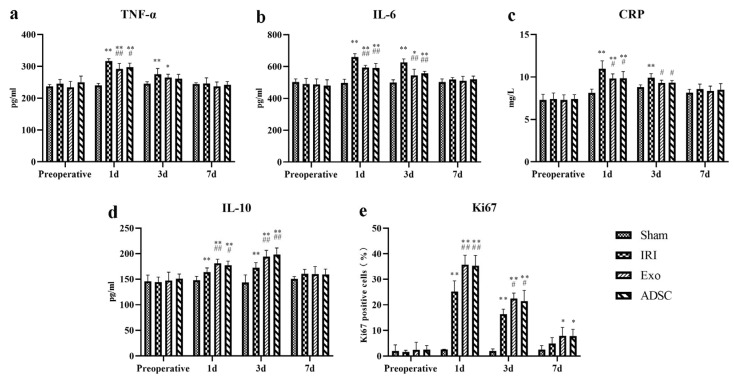
The TNF-α (**a**), IL-6 (**b**), CRP (**c**), and IL-10 (**d**) levels in the serum. Quantitative analysis of Ki67-positive cells (**e**). The results are presented as mean ± standard deviations (SD), * *p* < 0.05, ** *p* < 0.01 versus the Sham group, ^#^ *p* < 0.05, ^##^ *p* < 0.01 versus the IRI group.

**Figure 4:**
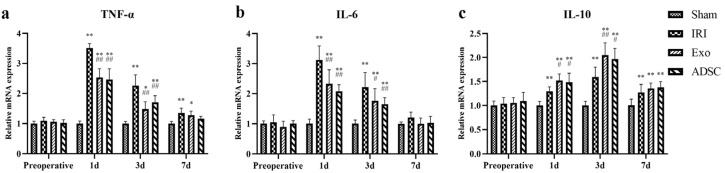
The mRNA expression of TNF-α (**a**), IL-6 (**b**), and IL-10 (**c**) in the liver. The results are presented as mean ± SD, * *p* < 0.05, ** *p* < 0.01 versus the Sham group, ^#^ *p* < 0.05, ^##^ *p* < 0.01 versus the IRI group.

After liver injury, the serum levels of the pro-inflammatory cytokines TNF-α, IL-6, and CRP levels (Figure 3a–c) were significantly elevated (1d: *p* < 0.01 or *p* < 0.05 vs. Sham group) in the IRI, Exo, and ADSC groups and peaked on day 1 post surgery. On the third day post-operation, CRP levels had already normalized in Exo and ADSC groups on day three post-operation (*p* > 0.05 vs. Sham group), while the IRI group still showed significantly elevated levels of these factors (*p* < 0.01 vs. Sham group), which only normalized on the seventh day post-operation. The Exo group and ADSC group exhibited significantly lower levels of these indicators compared to the IRI group (1d, 3d: *p* < 0.01 or *p* < 0.05). The serum levels of the anti-inflammatory cytokine IL-10 (Figure 3d) were also significantly elevated in the IRI, Exo, and ADSC groups than those in the Sham group (1d, 3d: *p* < 0.01 or *p* < 0.05) and peaked on the third day post-liver injury. The Exo and ADSC groups showed significantly higher levels of IL-10 compared to the IRI group on days 1 and 3 post-operation (1d and 3d: *p* < 0.01 or *p* < 0.05). The expressions of TNF-α, IL-6, and IL-10 mRNAs in the liver tissue were consistent with the changes in serum levels (Figure 4). Taken together, ADSCs and ADSCs-exo interventions can control the inflammatory response after liver injury in minipigs.

### 2.4. ADSCs-exo Promoted Liver Regeneration after Injury

**Figure 5:**
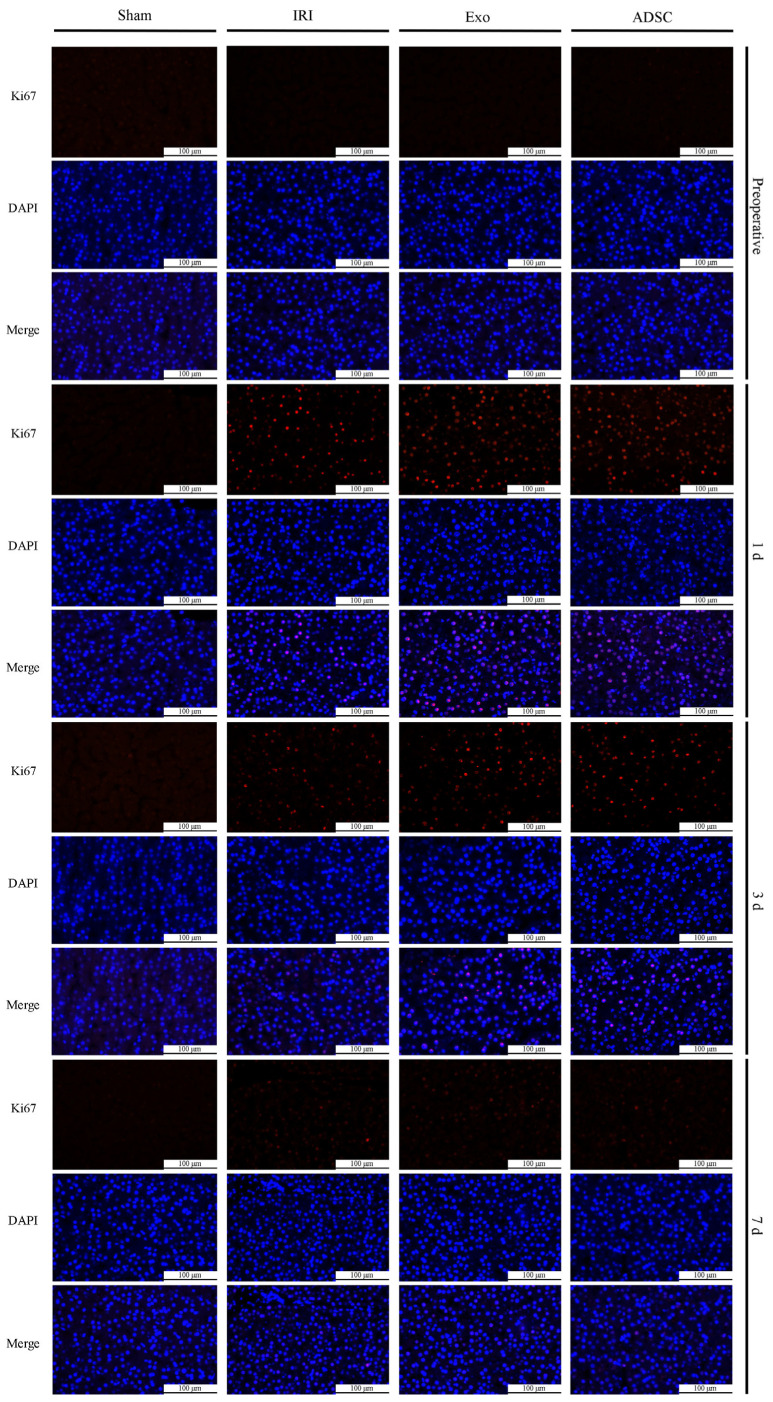
Ki67 staining results. The red color indicates Ki67-positive nuclei and the blue color indicates normal nuclei.

**Figure 6:**
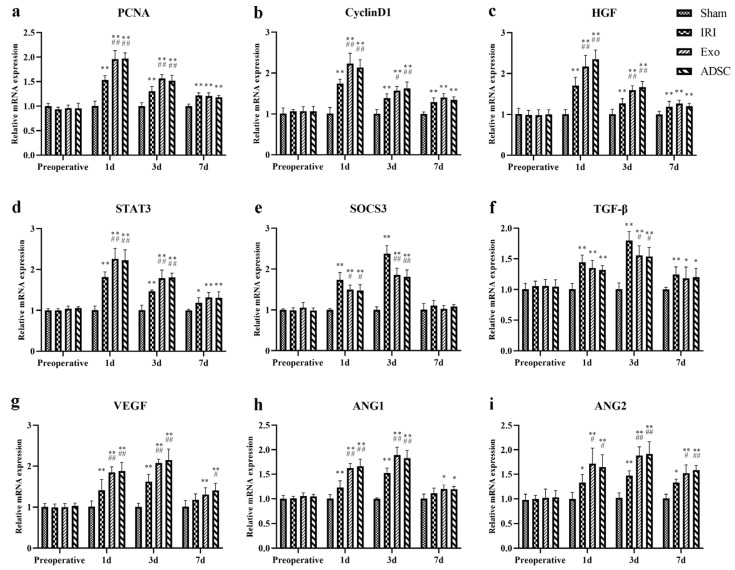
The mRNA expression of PCNA (**a**), Cyclin D1 (**b**), HGF (**c**), STAT3 (**d**) SOCS3 (**e**), TGF-β (**f**), VEGF (**g**), ANG1 (**h**) and ANG2 (**i**) in the liver. The results are presented as mean ± SD, * *p* < 0.05, ** *p* < 0.01 versus the Sham group, ^#^ *p* < 0.05, ^##^ *p* < 0.01 versus the IRI group.

**Figure 7:**
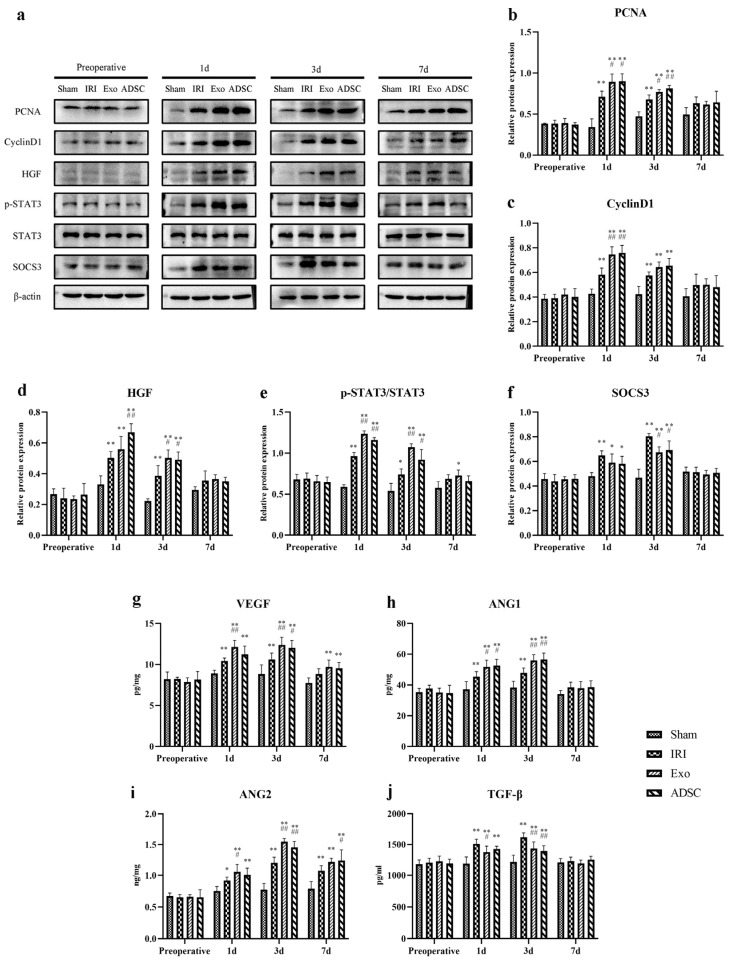
Representative Western blot analysis of PCNA, CyclinD1, HGF, p-STAT3, STAT3 and SOCS3 (**a**) in the liver. Quantification of PCNA (**b**), CyclinD1 (**c**), HGF (**d**), p-STAT3/STAT3 (**e**) and SOCS3 (**f**). The cytokine levels of VEGF (**g**), ANG1 (**h**), ANG2 (**i**) and TGF-β (**j**) in the liver. The results are presented as mean ± SD, * *p* < 0.05, ** *p* < 0.01 versus the Sham group, ^#^ *p* < 0.05, ^##^ *p* < 0.01 versus the IRI group.

Ki67 staining was used to detect proliferating cells in the liver tissues. As shown in Figure 5, the Ki67-positive cells in the IRI, Exo, and ADSC groups increased as early as the first day after liver injury. The quantitative analysis showed in Figure 3e that the percentage of Ki67-positive proliferative cells significantly increased (*p* < 0.01 vs. Sham group) on the first day after liver injury in the IRI, exo, and ADSC groups, which declined on day three but still remained significantly elevated (3d: *p* < 0.01, 7d: *p* < 0.05 vs. Sham group). Compared to the IRI group, the percentage of Ki67-positive cells was significantly higher in the Exo and ADSC groups on days 1 and 3 (*p* < 0.01 or *p* < 0.05). In addition, both the expressions of mRNAs and protein levels of PCNA and CyclinD1 were consistent with the results of Ki67 (Figure 6a,b and Figure 7a–c).

To further explore the regeneration of hepatocytes and sinusoidal endothelial cells, we analyzed the mRNA expression levels of relevant factors. HGF and STAT3 (Figure 6c,d), which are associated with hepatocyte regeneration, were significantly elevated in the IRI, Exo, and ADSC groups after liver injury (1d, 3d: *p* < 0.01 vs. Sham group). VEGF, ANG1, and ANG2 (Figure 6g–i), which are related to sinusoidal endothelial cell regeneration, also showed significant upregulation in the IRI, Exo, and ADSC groups (1d, 3d: *p* < 0.01 vs. Sham group). Moreover, relatively high levels of these factors were sustained 7 days post-surgery. HGF and STAT3 peaked on day one post-surgery, whereas the peak expression levels of VEGF, ANG1, and ANG2 were detected on the third day after surgery. As expected, compared to the IRI group, the Exo and ADSC groups significantly upregulated these factors (1d, 3d: *p* < 0.01 or *p* < 0.05), and ANG2 levels remained significantly higher in the Exo and ADSC groups on day 7 (*p* < 0.01 or *p* < 0.05). The negative regulators of liver regeneration, TGF-β, and SOCS3 (Figure 6e,f) also exhibited a significant increase in expression levels after liver injury in the IRI, Exo, and ADSC groups (1d, 3d: *p* < 0.01) and peaked on day 3 post-operation. However, the Exo and ADSC groups showed significantly lower levels of TGF-β (3d: *p* < 0.05) and SOCS3 (1d: *p* < 0.05, 3d: *p* < 0.01) compared to the IRI group. We also detected the protein expression of the pro-regenerative indicators (HGF, p-STAT3/STAT3, and SOCS3) (Figure 7a,d–f) and cytokines (VEGF, ANG1, ANG2, and TGF-β) (Figure 7g–j) in the liver tissues, which were generally consistent with that of the mRNAs.

Taken together, ADSCs and ADSCs-exo play a crucial role in pro-regenerating hepatocyte and sinusoidal endothelial cells after liver injury in minipigs.

### 2.5. ADSCs-exo Has a Similar Pro-Regenerative Capacity to ADSCs after Liver Injury

The histopathological changes in hepatic cord structures (Figure 1) and liver sinusoids, as well as the extent of hemorrhage, inflammatory infiltration, and hepatocyte swelling, were similar in the Exo and ADSC groups at the first day after liver injury. Only hepatocyte swelling was slightly milder in the ADSC group than that in the Exo group 3 days after surgery. The hepatocyte nuclei density and rearrangement were eventually at the same behavior both intervention groups on day 7 post surgery. Furthermore, on the liver function, ALT, TBIL, and HA showed slight differences between the Exo and ADSC groups but were not statistically significant (Figure 2). In addition, the expression levels of the inflammatory factors in serum and liver tissues (Figure 3a–d and Figure 4), and those of factors related to liver regeneration (Ki67, PCNA, CyclinD1, STAT3, SOCS3, and ANG1) (Figure 3e, Figure 5, Figure 6 and Figure 7), were also similar between the Exo and ADSC groups. Despite the Exo group showing a slight advantage in the levels of VEGF, ANG2, and TGF-β cytokines, while the protein level of HGF was better in the ADSC group on day 1 post-surgery, all of them were not statistically significant (*p* > 0.05). Taken together, within the scope of this study, ADSCs-exo and ADSC interventions could achieve similar therapeutic effects on liver injury in minipigs.

## 3. Discussion

Ischemia and reperfusion of the liver lead to a decrease in transmembrane potential, which causes cell swelling, vacuolization, and even loss of membrane integrity, resulting in an inflammatory response [26,27]. The reduction in liver volume after hepatectomy and the significant increase in the shear force of blood flow in the hepatic sinusoids aggravate cellular injury [4]. Liver function and the extent of liver injury can be evaluated by measuring the serum levels of certain biochemical markers. For instance, ALT is leaked from the injured liver cells into the bloodstream due to the altered membrane permeability. Furthermore, severely swollen liver cells exhibit impaired bilirubin uptake, binding, and conversion, which can impede the biliary excretion of bilirubin, and increase TBIL levels in the bloodstream.

However, when the liver loses a significant amount of functional liver volume due to injury, it rapidly initiates tissue regeneration [28]. The resulting increase in shear force, as well as the growth factors and cytokines secreted during the inflammatory response, trigger liver regeneration [29]. TNF-α, IL-6, and IL-10 are primarily secreted by the non-parenchymal liver cells, and they promote liver regeneration by activating NF-κB and STAT3 transcription in the hepatocytes [30,31,32]. HGF binds to the specific receptor cMet on the surface of hepatocytes through paracrine or endocrine pathways and initiates transcription effects [33]. Furthermore, HGF, IL-10, IL-6, and JAK/STAT signaling pathways promote the transition of the hepatocytes from the G0 to G1 stage in the regenerating liver [34]. CyclinD1 serves as a marker for hepatocytes breaking through the G1 phase and entering the S phase [35]. Ki67 and PCNA are nuclear antigens associated with proliferating cells, expressed in cells undergoing proliferation, and reflecting the extent of cell proliferation [36].

Liver regeneration also entails proliferation of the non-parenchymal cells, as well as reconstruction of the hepatic lobules [37,38]. VEGF is the most potent pro-angiogenic factor, and its production in the liver cells is stimulated by hypoxia and increased shear forces. VEGF induces endothelial cell proliferation by binding to the surface receptors [39]. ANG1 is produced by perivascular support cells through paracrine action and promotes endothelial cell budding and tube formation, thereby stabilizing endothelial cell activity. On the other hand, ANG2 is mainly synthesized by sprouting vascular endothelial cells and competitively inhibits excessive angiogenesis through autocrine action, thus maintaining a balance between angiogenesis and regression. Interestingly, ANG2 exhibits pro-angiogenic effects in the presence of VEGF, and anti-angiogenic effects in its absence [40]. HA is a marker of hepatic sinusoidal endothelial cell function. Nearly all HA is transported through the bloodstream by the sinusoidal endothelial cells to the liver for clearance and metabolism. However, dysfunctional sinusoidal endothelial cells exhibit diminished phagocytic and metabolic capacity for HA, resulting in the accumulation of HA in the serum [41].

Once the regenerating liver achieves adequate size and sufficient functionality, anti-proliferative signals are initiated to terminate liver regeneration. TGF-β is secreted by both parenchymal and non-parenchymal cells of the liver and can terminate hepatocyte over-proliferation by downregulating specific genes [42]. In addition, TGF-β regulates the interaction between hepatic cells and the extracellular matrix (ECM) by promoting the synthesis of ECM components, which promotes the reconstruction of liver lobules. SOCS3, a negative feedback regulator of liver regeneration, is induced by IL-6 to inhibit STAT3 phosphorylation [43].

The regulatory role of MSCs-exo in liver regeneration has been validated in rodent and cellular models. Tan et al. demonstrated that MSCs-exo promoted liver regeneration in a mouse model of CCl4-induced liver injury and enhanced proliferation of acetaminophen and H2O2-treated hepatocytes in vitro by upregulating CyclinD1 and PCNA via the HGF/c-Met and STAT3 signaling pathways [44]. Likewise, Rong et al. showed that bone marrow-derived MSCs-exo promoted liver regeneration in the CCl4-induced rat liver fibrosis model by upregulating CyclinD1 and inhibiting IL-6 secretion [45]. In a study conducted by Jin et al., ADSCs-exo alleviated D-Gal or D-Gal/LPS-induced acute liver failure in a rat model by promoting hepatocyte proliferation through the HGF/cMet/STAT3 pathway and improving the survival rate [46]. Furthermore, Anger et al. found that bone marrow-derived MSCs-exo reduced ALT and AST levels, increased the percentage of Ki67-positive hepatocytes, and suppressed transcription of inflammation-related genes in a mouse model of hepatic IRI [47]. In addition, Ichinohe et al. found that bone marrow-derived MSCs-exo containing miR-146a-5p stimulated hepatic progenitor cells and promoted liver regeneration in a rat model of liver hepatectomy [48]. Piao et al. similarly showed that ADSCs-exo promoted liver regeneration after hepatectomy combined with hepatic IRI in a rat model by upregulating VEGF and CyclinD1 [49].

Our study is the first to provide evidence of the liver regenerative effects of ADSCs-exo in a large animal model. We found that ADSCs-exo intervention reduced ALT, TBIL, and HA levels in minipigs after liver injury, suppressed the early pro-inflammatory factors TNF-α and IL-6, and upregulated the late anti-inflammatory factor IL-10. ADSCs-exo also promoted hepatocyte proliferation by activating the HGF and STAT3 pathways and stimulated sinusoidal endothelial cell proliferation by upregulating VEGF and ANG. Moreover, ADSCs-exo downregulated the negative regulatory molecules TGF-β and SOCS3 in the early stages following liver injury, thereby facilitating the repair and regeneration of the residual liver tissue.

The therapeutic effect of ADSCs-exo was comparable to that of ADSCs and may be attributed to a similar mode of action. Piao et al. also demonstrated similar regenerative effects of ADSCs-exo and ADSCs and identified the same underlying mechanism of action [49]. Consistent with our findings, Rostom et al. showed that MSCs-exo and MSCs had similar therapeutic effects in rats with CCl4-induced liver injury [50]. Furthermore, one group isolated MSCs-exo and MSCs-exo-free secretomes and found that MSCs-exo, rather than the secretome lacking MSCs-exo, alleviated acute liver injury and promoted regeneration [51]. These findings suggest that the paracrine effects of MSCs are mediated by MSCs-exo.

MSCs-exo contain pro-regenerative cytokines such as ICAM-1, ANG2, and IL-6 [52], anti-inflammatory factors such as IL-10 [53], and biologically active miRNAs such as miR-146a-5p [48], miR-182-5p [54], and miR-20a [55]. However, we did not characterize the proteins and genes of the ADSCs-exo isolated in this study. Nevertheless, previous transcriptomics and proteomics analyses of porcine ADSCs-exo and ADSCs have revealed significant overlap of RNAs and proteins associated with various pathways, albeit with some differences in their expression [56,57]. This may also explain the slight differences we observed in the expression of HGF, VEGF, ANG2, and TGF-β between the Exo and ADSC groups after liver injury.

Kupffer cells are the first cells to be activated during hepatic IRI and one of the main sources of pro-inflammatory factors [58]. In addition, MSCs-exo are primarily internalized by Kupffer cells rather than liver cells [54,59]. Therefore, the pro-regenerative effects of MSCs-exo on hepatocytes and sinusoidal endothelial cells are likely mediated by the cytokines produced by Kupffer cells. Based on their phenotypes and cytokine profiles, Kupffer cells can be classified into the M1 and M2 populations. M1-type Kupffer cells secrete pro-inflammatory cytokines including IL-6 and TNF-α, while M2-type Kupffer cells produce anti-inflammatory cytokines such as IL-10 and TGF-β [60]. The regulatory effect of MSCs-exo on macrophage polarization has been reported in many studies. Zhao et al. demonstrated that bone marrow-derived MSCs-exo promoted macrophage polarization towards the M2 phenotype by inhibiting the activity of TLR4 in a rat model of myocardial IRI injury, which reduced the infarct size and suppressed the inflammatory response [61]. Li et al. showed that induced pluripotent stem cell-derived exosomes (iPSCs-exo) facilitated neural regeneration in mouse models of neural injuries by promoting the polarization of M1 macrophages to the M2 phenotype through miR-199b-5p-mediated inhibition of HGF [62]. Given the effect of ADSCs-exo on inflammatory factors, we can surmise that ADSCs-exo may inhibit the M1-type polarization of Kupffer cells in the early stages and promote the M2-type phenotype in the later stages of liver regeneration. However, the reciprocal regulation of macrophage polarization and liver regeneration by ADSCs-exo needs further experimental validation and will be the focus of our next study.

## 4. Materials and Methods

### 4.1. Preparation of ADSCs and ADSCs-exo

ADSCs and ADSCs-exo were obtained and identified as described in a previous article [63]. In brief, minipigs were anesthetized, and the inguinal fat tissue was excised. The extracted fat tissues were digested with type I collagenase to isolate the ADSCs, which were subsequently cultured for 4–5 passages and then characterized and collected. ADSCs-exo were obtained from ADSCs cultured in a serum-free medium for 36 h via ultrafiltration and ultracentrifugation.

### 4.2. Animals

The Animal Ethics Committee of Northeastern Agricultural University approved all the experiments conducted in this study. Healthy Bama minipigs (*n* = 24, aged 4–5 months, half male and half female, and weighing 20–30 kg) were randomly divided into the following four groups: Sham, IRI, Exo, and ADSC groups (*n* = 6 per group). The animals in the Sham group underwent general anesthesia, establishment of the surgical access with pneumoperitoneum, and closure of the surgical incision. The animals in the IRI group underwent laparoscopic hepatectomy combined with IRI. The modeling procedure for the Exo and ADSC groups was the same as that for the IRI group.

The following interventions were applied via the portal vein immediately after the liver resection:

Sham group: 5 mL of phosphate-buffered saline (PBS) was administered.

IRI group: 5 mL of PBS was administered.

Exo group: 5 mL of PBS containing 5 × 10^9^ particles/kg of ADSCs-exo was administered.

ADSC group: 5 mL of PBS containing 1 × 10^6^ cells/kg of ADSCs was administered.

### 4.3. Surgical Procedure of Laparoscopic Partial Hepatectomy Combined with IRI

The minipigs were anesthetized using propofol and maintained by isoflurane through endotracheal intubation. For the surgical access, a 10 mmHg pneumoperitoneum was established using the four-trocar technique after routine sterilization. The liver ligaments were dissected to achieve full mobilization of the liver lobes. The gallbladder duct and artery were separated, and tourniquets were used to effectively block the inflow of the right hepatic blood supply. Consequently, the liver parenchyma transitioned from its normal reddish-brown color to a yellowish-brown hue, and a distinct demarcation line of ischemia appeared between the left and right hepatic lobes. After maintaining the ischemia in the right hepatic lobe for 60 min, the tourniquets were removed, and the right hepatic lobe gradually regained its red color as the blood supply was restored. Subsequently, the inflow and parenchyma of the left hepatic lobe were ligated, and the left liver lobe was resected. Following hepatectomy, corresponding interventions were slowly administered through the portal vein. After ensuring no bleeding or bile leakage at the injection site and cutting surface, the abdominal cavity was rinsed with saline, and the excised left liver lobe was put in a specimen bag. Then, the pneumoperitoneum was released, and the trocar ports were sutured. Subsequently, the animals were awakened in a warm and quiet environment and received postoperative pain management.

Blood samples were collected through the anterior vena cava before the surgery and on postoperative days 1, 3, and 7. Additionally, liver samples were collected using minimally invasive laparoscopy at the same time.

### 4.4. Liver-Function Analysis

Blood samples were centrifuged at 300× *g* for 15 min, and the alanine aminotransferase (ALT) and total bilirubin (TBIL) levels in the serum were measured using a blood biochemistry analyzer (TBA-2000FR, Canon Medical. Systems Corporation, Tochigi, Japan).

### 4.5. Histopathological Assessment

Fresh liver samples were minced into 1 cm^3^ pieces, fixed in 4% paraformaldehyde, embedded in paraffin, and then sectioned at 4–5 μm thickness. Subsequently, hematoxylin-eosin (HE) staining was performed following standard protocols, and histopathological changes were observed under a light microscope.

### 4.6. Enzyme-Linked Immunosorbent Assay (ELISA)

Serum levels of tumor necrosis factor-α (TNF-α), interleukin-6 (IL-6), C-reactive protein (CRP), interleukin-10 (IL-10), hyaluronic acid (HA), and transforming growth factor-β (TGF-β), and hepatic levels of vascular endothelial growth factor (VEGF), angiopoietin 1 (ANG1), and ANG2 were measured using specific ELISA kits according to the instructions of the manufacturer (Mlbio, Shanghai, China).

### 4.7. Ki67 Immunofluorescence Staining

Each group was randomly sampled with 3 samples. The sections were deparaffinized, and antigen retrieval was performed via microwave-heating in a 0.01 M sodium citrate buffer (Beyotime, Shanghai, China). Membrane permeabilization was achieved using 0.25% Triton X-100 (Beyotime, Shanghai, China), and blocking was carried out using 10% goat serum (Beyotime, Shanghai, China). The sections were incubated with an antibody against Ki67 (1:100, Novus, NB500-170SS, Centennial, CO, USA) in a humid chamber overnight at 4 °C. After CY3 fluorescent secondary antibody (1:500, Abclonal, AS007, Wuhan, China) labeling, the samples were mounted with a DAPI-containing anti-fade mounting medium (Biosharp, Hefei*,* China). Subsequently, they were observed and photographed using a fluorescence microscope (Leica, Wetzlar, Germany). Ki67-positive nuclei were counted in five randomly selected fields per sample. The regenerative cell index was calculated as the ratio of Ki67-positive cells to the total number of cells.

### 4.8. Reverse Transcription–Quantitative Polymerase Chain Reaction (RT-qPCR)

Total RNA was extracted from liver samples by using the TRIzol reagent according to the instructions of the manufacturer (Invitrogen, Carlsbad, CA, USA). Subsequently, the extracted RNA was reverse-transcribed into cDNA (Vazyme, Nanjing, China). The cDNA samples were labeled with the SYBR Green I fluorescent dye, and amplified using a three-step method according to the instructions of the manufacturer (Innovagene, Changsha, China) in a LightCycler 480 instrument (Roche, Basel, Switzerland). The relative gene-expression levels were determined using the 2^−ΔΔCt^ method, with β-actin as the reference gene. The primers used for this process were synthesized by UW Genetics and are detailed in Table 1.

### 4.9. Western Blotting

For western blotting, each group was randomly sampled with 3 samples. Liver samples were lysed using RIPA buffer (Beyotime, Shanghai, China) containing 1% protease inhibitor (Beyotime, Shanghai, China) and 1% phosphatase inhibitor (Merck, Darmstadt, Germany). The protein concentration was determined using the BCA assay (Beyotime, Shanghai, China) and normalized against that of saline. Subsequently, the samples were denatured by boiling in the loading buffer (Beyotime, Shanghai, China) and subjected to SDS-PAGE (LEAGENE, Beijing, China). The protein bands were transferred onto NC or PVDF membranes (Pall, Washington, DC, USA) via the wet-transfer method. After blocking the membranes with 5% fat-free milk or 5% BSA at room temperature for 2 h, they were incubated overnight at 4 °C with antibodies against PCNA (1:2000, CST, 2586T, Beverly, MA, USA), CyclinD1 (1:1000, Immunoway, YT1172, Plano, TX, USA), HGF (1:500, Santa Cruz, SC-374422, Dallas, TX, USA), STAT3 (1:1000, Immunoway, YT4443, Plano, TX, USA), p-STAT3 (1:1000, Immunoway, YP0251, Plano, TX, USA), SOCS3 (1:1000, Immunoway, YM1098, Plano, TX, USA), and β-actin (1:40,000, Proteintech, 81115-1-RR, Wuhan, China). The following day, the membranes were incubated with secondary antibodies at room temperature for 2 h. Target protein bands were visualized using ECL (meilunbio, Dalian, China) and the Tanon 5200 system, and their average optical density was determined using ImageJ 1.52a (Java 1.8.0_112 64-bit).

### 4.10. Statistical Analysis

Statistical analysis was performed using SPSS 22.0. The data are presented as mean ± SD. Group comparisons were carried out using one-way ANOVA followed by LSD tests, with the statistical significance set at *p*-values < 0.05.

## 5. Conclusions

ADSCs-exo significantly promoted liver regeneration in minipigs after partial hepatectomy combined with hepatic IRI by enhancing the proliferation of hepatocytes and sinusoidal endothelial cells, most likely through the regulation of macrophage polarization. Our findings provide experimental evidence for the regenerative potential of ADSCs-exo in the liver, along with a foundation for cell-free liver regenerative therapies. In addition, our study validates the significant role of ADSCs-exo in the paracrine effects of ADSCs.

## Figures and Tables

**Table 1:** Gene-specific primers used for RT-qPCR.

Gene	Forward Primer Sequence (5′-3′)	Reverse Primer Sequence (5′-3′)
PCNA	GGCTCTATCCTGAAGAAGGTGCTG	GACATGAGACGAGTCCATGCTCTG
CyclinD1	AAGTGCGTGCAGAAGGAAAT	AGGAAGCGGTCCAGGTAGTT
HGF	TGATCAACTCAGACGGCCTA	AGCCCCAGCACATATTTCAG
STAT3	GTGGAGAAGGACATCAGCGGTAAG	AGGTAGACCAGCGGAGACACAAG
SOCS3	GGTCACCCACAGCAAGTTTCCC	TCCAGTAGAAGCCGCTCTCCTG
VEGF	CATGGCAGAAGGAGACCAGAAACC	CACAGGACGGCTTGAAGATGTACTC
ANG1	AAATGGAGGGGAAGCACAAGGAAG	ACTGTTATTGGTGGTGGCTCTGTTC
ANG2	CACCTACACGCTGACCTTTCCTAAC	CGCTGAATAACTGTCCATCCACCTC
TGF-β	CCATTCGCGGCCAGATT	GCTCCGGTTCGACACTTTC
β-actin	TCTGGCACCACACCTTCT	TGATCTGGGTCATCTTCTCAC

## Data Availability

The data used to support the findings of this study are available from the corresponding author upon request.

## Abbreviations

ADSCs	Adipose-derived mesenchymal stem cells
ADSCs-exo	Exosomes from adipose-derived mesenchymal stem cells
ALT	Alanine aminotransferase
ANG	Angiogenin
CRP	C-Reactive Protein
HA	Hyaluronic acid
HGF	Hepatocyte growth factor
IRI	Ischemia/reperfusion injury
IL	Interleukin
MSCs	mesenchymal stem cells
MSCs-exo	MSCs-derived exosomes
NF-κB	Nuclear factor kappa B
PCNA	Proliferating cell nuclear antigen
STAT	Signal transducer and activator of transcription
SOCS3	Suppressor of cytokine signalling
TBIL	Total bilirubin
TGF	Transforming growth factor
TNF	Tumor necrosis factor
VEGF	Vascular endothelial growth factor
